# Prevalence of biofilm formation and vancomycin-resistant genes among *Enterococcus faecium* isolated from clinical and environmental specimens in Lorestan hospitals

**Published:** 2018-04

**Authors:** Mansour Goudarzi, Ashraf Mohabati Mobarez, Shahin Najar-Peerayeh, Mohsen Mirzaee

**Affiliations:** 1 Department of Medical Bacteriology, Faculty of Medical Sciences, Tarbiat Modares University, Tehran, Iran; 2 Department of Laboratory Sciences, Borujerd Branch, Islamic Azad University, Borujerd, Iran

**Keywords:** *Enterococcous faecium*, Antibiotic resistance, Vancomycin, Biofilm formation

## Abstract

**Background and Objectives:**

The antibiotic resistance among *Enterococcus faecium* strains has increased worldwide. Additionally, biofilm-forming isolates of *E. faecium* play an important role in human infections. This study was conducted to investigate the prevalence of virulence and antibiotic resistance genes between biofilm-producing and non-biofilm-producing *E. faecium* strains.

**Materials and Methods:**

In this study, 228 *E. faecium* isolates from clinical and environmental specimens were obtained from different wards of hospitals in Lorestan province (Iran). Then, the pattern of antibiotic resistance and minimum inhibitory concentration (MIC) against β-lactams, glycopeptides, aminoglycosides and other common antibiotics was investigated using disk diffusion and agar dilution methods. Biofilm formation was investigated using polystyrene microtiter plates. PCR assay was conducted for antibiotic resistance and biofilm related genes. Pulse field gel electrophoresis (PFGE) was used to determine the clonal spread of isolates.

**Results:**

Most of isolates (78%) were resistant to penicillin, but all were susceptible to linezolid and tigecycline. The biofilm-producing isolates were more resistant to β-lactams, glycopeptides and aminoglycosides compared to non-biofilm-producing strains. In biofilm-producing isolates, *pilA, pilB, efaAfm* and *esp* were the dominant virulence genes and *vanA* and *pbp5* genes were the dominant resistant genes. PFGE analysis exhibited a similar pattern between the clinical and environmental isolates, suggesting the presence of a common origin of the infection by *E. faecium*.

**Conclusion:**

The results of the antibiotic resistance, biofilm assay, and PFGE analysis suggest that there is a common clone of persistent and biofilm-producing strains of *E. faecium*, which could rapidly disseminate in patients and the environment.

## INTRODUCTION

Enterococci are a major class of hospital-acquired pathogens and show resistance to several antibiotics, specifically vancomycin. Most vancomycin-resistant enterococci (VRE) belong to the species *E. faecium*, a major agent in hospital-acquired infections (1). Enterococci are intrinsically resistant to many antibiotics and are able to acquire drug resistance either by chromosomal mutations, transfer of plasmids, or transposon acquisition containing genetic sequences that confer resistance (2). Biofilms are believed to be an important factor in the pathogenesis of enterococcal infections, along with other virulence factors, such as cytolysin, gelatinase, serine protease, hyaluronidase, aggregation substance, extracellular surface protein, and cell wall adhesins. Around 80% of persistent bacterial infections in the United States are associated with biofilms (3). Biofilm formation in enterococci is complex and multifactorial, and the involvement of various bacterial virulence factors in this process is still unclear. Several genes have been found to be important in the biofilm formation, such as *esp* and *fsr* through their effect on gelatinase and aggregation substance (4). The present study aimed at investigating the prevalence of biofilm formation and vancomycin-resistant genes among *Enterococcus faecium* isolated from clinical and environmental specimens in Lorestan hospitals.

## MATERIALS AND METHODS

### Bacterial isolate.

From August 2014 to February 2015, a total of 690 *Enterococci* isolates were collected from 2 main hospitals (Shariati and Shahid-Chamran hospitals) in city of Borujerd (Lorestan province, Iran). Overall, 188 clinical and 40 environmental samples were collected. The clinical isolates were collected from urine, wound, blood, stool, intravenous catheter, and trachea. Environmental samples were collected from patients’ bathrooms, beds, and tables and staff’s bathrooms and tables, as well as ventilators and oxygen pumps in the patients’ rooms. All *Enterococci* isolates were identified according to their genus and species levels by Gram staining, catalase reaction, growth in 6.5% NaCl, motility assessment, use of arabinose, bile and esculin hydrolysis, and also by pigment production after their growth on enterococcus selective agar (BBL, USA), all based on Facklam and Collins criteria (5). In addition, the isolates were identified with PCR using specific primers for the amplification of *E. faecium ddl* gene (6).

### Antimicrobial susceptibility testing.

Antimicrobial susceptibility test was performed by the disk diffusion method and according to the Clinical and Laboratory Standards Institute (CLSI) guidelines for 12 antimicrobial agents including penicillin (10 μg), ampicillin (10μg), gentamicin (10μg), streptomycin (10 μg), chloramphenicol (30μg), erythromycin (15μg), tetracycline (30μg), nitrofurantoin (300μg), ciprofloxacin (5μg), rifampin (5μg), linezolid (30μg), tigecycline (15μg), and vancomycin (30μg) (Padtan Teb, Tehran, IRAN).

### Minimum inhibitory concentrations (MICs).

The MICs for vancomycin, ampicillin, and gentamicin were determined by the agar dilution method. The results were interpreted according to guidelines from the CLSI (7).

### Biofilm formation assay.

All 228 isolates of *E. faecium* were assayed for their ability to form biofilms. Biofilm formation was tested on polystyrene microtiter plates, and the optical density results were interpreted as described previously. First, 180 μL of trypticase soy broth (Becton Dickinson), supplemented with 1.5% glucose, was added to each well of a sterile 96-well polystyrene microtiter plate, and then 20 μL of bacterial suspension was added. The plates were incubated for 24 hours at 35 ± 2°C under static conditions. After incubation period, the media was removed, and the wells were washed 3 times with sterile saline. Next, the adherent bacteria were fixed with methanol for 20 minutes, stained with 0.5% crystal violet for 15 minutes, and biofilm was eluted with ethanol for 30 minutes. Absorbance was measured at 492 nm using an Expert Plus microtiter plate reader (ANTOS 2020). *Staphylococcus epidermidis* ATCC 35984 was the positive control strain. The cut-off calculation was used to interpret the test results based on the following formula: Cutoff = (mean (OD_492_) ±3SD (OD_492_)) + OD_492_ (Blank)). Strains with OD_492_≤ 0.45, 0.45 – 0.55 and ≥ 0.55 were considered as non-adherent, weakly adherent, and strongly adherent, respectively (8, 9).

### DNA extraction.

DNA extraction was performed using Cinnapure TMDNA extraction kit (Cinnagen, Iran). Bacterial pellet was suspended in 100 μL G+ pre-lysis buffer and added to 20 μL lysozyme and incubated at 37°C for at least 30 minutes. After adding lysis buffer and precipitation solution, the solution was transferred to a spin column, where DNA was washed and eluted by elution buffer at 65°C (10).

### PCR amplification of resistance and biofilm formation genes.

For PCR amplification assay, specific primers of *E. faecium ddl* gene, vancomycin resistance genes (*vanA* and *vanB*), β-lactam resistance genes (*pbp5, pbpZ* and *blaZ*), and biofilm formation genes (*asa1, pilA, pilB, hyl, esp, fsr, efaA_fm_, gelE*) were used as described previously (11). Polymerase chain reaction product sizes for virulence genes (*asa1* (530bp), *pilA* (459bp), *pilB* (959bp), *hyl* (275bp), *esp* (419bp), *fsr* (218bp), *gelE* (210bp), and *efaA_fm_* (199bp)) and resistance genes (*vanA* (734bp), *vanB* (420bp), *pbp5* (65bp), *pbpZ* (1231bp), and *blaZ* (861bp)) are demonstrated in Tables 2 and 3. *E. faecium* ATCC 51559 and *E. faecalis* ATCC 51229 standard strains carrying the *vanA* and *vanB* genes were used (12). The PCR products were analyzed on 1% agarose gel, stained with safe stain, and visualized under UV light after the electrophoresis.

**Table 2:** The rate (%) virulence genes of *E. faecium* strains isolated from clinical and environmental sample

**Origin of isolates (n) (%)**	**Virulencegenes (n) (%)**							

	***pilA***	***pilB***	***efaA_fm_***	***esp***	***hyl***	***asa1***	***gelE***	***fsr***
Urine (45) (38%)	45	42	43	41	19	19	7	2
Stool (16) (14%)	16	13	14	14	8	6	4	1
Wound (11) (10%)	10	9	9	10	4	2	3	1
Blood (10) (9%)	9	9	8	7	5	3	3	0
Trachea (3) (2%)	3	3	3	2	3	1	2	1
Catheter (2) (2%)	2	1	1	1	1	1	1	0
Environment (30) (25%)	26	27	26	24	11	6	12	12
Total (117) (100%)	111 (94%)	106 (91%)	104 (88%)	99 (84%)	51 (41%)	38 (32%)	32 (27%)	17 (14%)

**Table 3:** The rate (%) resistance genes of *E. faecium* strains isolated from clinical and environmental sample

**Origin of isolates (n) (%)**	**Resistance genes (n) (%)**						

	***vanA***	***van B***	***vanA&B***	***pbp5***	***pbpZ***	***blaZ***	**Biofilm formation**
Urine (45) (38%)	36	9	3	34	17	0	21 (18%)
Stool (16) (14%)	11	4	0	10	5	0	8 (7%)
Wound (11) (10%)	8	3	1	6	4	0	4 (4%)
Blood (10) (9%)	5	5	1	7	5	0	12 (11%)
Trachea (3) (2%)	3	0	0	3	3	0	1 (1%)
Catheter (2) (2%)	1	1	0	1	1	0	2 (2%)
Environment (30) (25%)	20	8	2	21	25	0	2 (2%)
Total (117) (100%)	84 (71%)	26 (22%)	7 (6%)	82 (70%)	60 (51%)	0 (0)	50 (45%)

### Pulsed-field gel electrophoresis (PFGE).

The genomic typing of isolates was performed by PFGE. Genomic DNA of isolates was provided in low melting agarose plugs according to a method presented by matushek et al. (13). *Enterococci* were grown overnight in 5 mL of brain heart infusion broth at 37°C. The cells were harvested and suspended in an equal volume of TE buffer containing 1M EDTA (PH = 9–9.3) and 1M Tris-Hcl (PH = 7.6). A portion (150μL) of this suspension was mixed with 150μL of 1.2% low-melting temperature agarose (Invitrogen, USA) in water at 40°C to 50°C and was then pipetted into a plug mold (Bio-Rad Laboratories, Richmond, Calif.) and was allowed to solidify. Then, plugs were incubated sequentially in the following solutions for the indicated times: 10 mL of lysis solution containing lysozyme at 0.5 mg/mL and DNase-free RNase at 10 mg/mL at 37°C for 24 hours with gentle shaking; 5 mL of sterile, ES buffer containing 1M EDTA (PH = 9–9.3); and Sarcosine 10% at 50°C for 1 hour and 10 mL of ESP solution containing proteinase K at 100 mg/mL and 1% sodium dodecyl sulfate at 50°C for 48 hours (13). The *Sma I* restriction enzyme (Roche, Manheim, Germany) was purchased to digest the DNAs in proper slices in agarose plugs. The plugs were placed in 1% aga-rose that was in 0.5% TBE and were electrophoresed with switch times ramped from 5 to 35 seconds at 6 V, with a run time of 23 hours at 14°C and an angle of 120 in the Bio-Rad CHEF-DRIII system (Bio-Rad, USA). *Salmonella* choleraesuis strain H9812 was used as a molecular size marker. Agarose plugs containing genomic DNA were digested with *XbaI* (Roche, Manheim, Germany) according to the manufacturer’s recommendations (13). After 24 hours, gels were stained with ethidium bromide and visualized under ultraviolet light. The banding patterns were clustered using weighted paired group (UPGMA) method by Gelcompar II software Version 4.0. Interpretation was done using the guidelines set out previously (14).

### Statistical analysis.

Chi-square test was performed for data analysis. P-values less than 0.05 were considered significant. Statistical analysis was done by SPSS. 21 Software.

## RESULTS

### Bacterial isolates.

Of 690 enterococcal isolates, 228 (33%) were identified as *E. faecium* and 439 (64%) as *E. faecalis*. The results were confirmed by PCR. On average, 37.8% of the *E. faecium* clinical isolates (n = 86) were cultured from urine specimens, followed by fecal samples 25.8% (n = 59); other results are presented in Table 1.

**Table 1:** The number and persentage of *E. faecium* isolates based on sampling origin

**Source of isolates**	**Values**
Urine	86 (37.8)
Stool	59 (25.8)
Wound	15 (6.7)
Blood	11 (4.8)
Trachea	13 (5.7)
Catheter	4 (1.7)
Environment	40 (17.5)
Total	228 (100)

### The antibiotic susceptibility pattern.

**Fig. 1:**
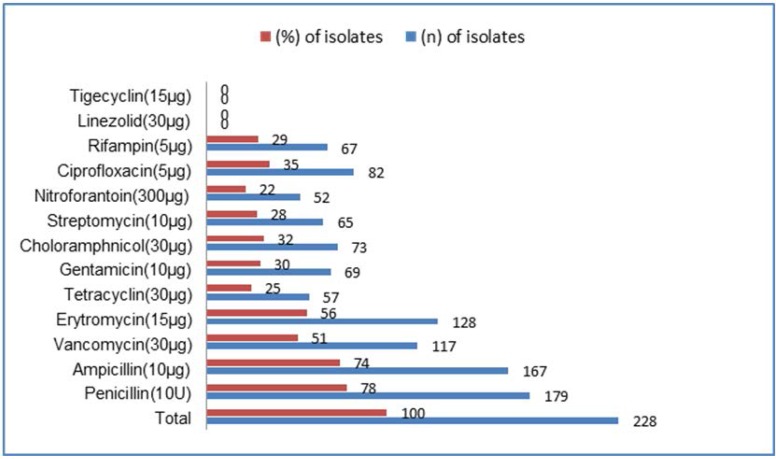
Antibiotic resistance of *E. faecium* isolate collected in this study based on disc diffusion concentration of disc presented after antibiotic names

Of the isolates, 51% were resistant to vancomycin (Fig. 1). Moreover, most of them were resistant to penicillin (78%) and ampicillin (74%). All the isolates were susceptible to tigecycline and linezolid.

### MIC determination.

**Fig. 2:**
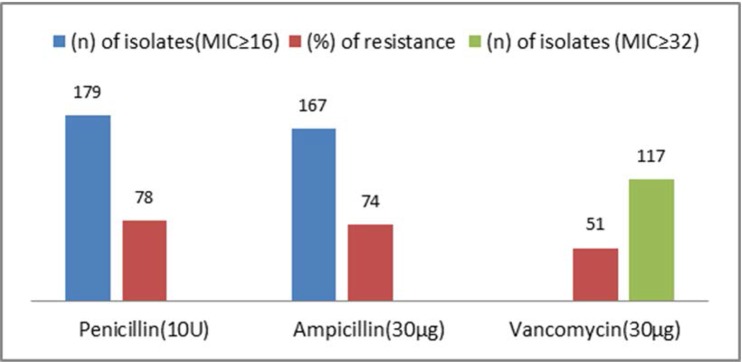
Antibiotic resistance of *E. faecium* isolates based on agar dilution method

MIC values for vancomycin, penicillin, and ampicillin ranged from 32 to 1024 μg/mL, 16 to 128 μg/mL, and 16 to 128 μg/mL, respectively (Fig. 2).

### Biofilm formation assay.

Quantitative assay of biofilm formation was conducted 3 times for each strain. Among 117 studied vancomycin resistance isolates, 57(49%) were adherent and 60 (51%) were non-adherent. Among 111 vancomycin sensitive isolates, 37 (33%) were adherent and 74 (67%) were non-adherent. Quantitative evaluation of biofilm formation revealed that 39 (45%) clinical isolates and 3 (9%) of all environmental isolates could produce biofilms.

### PCR amplification of biofilm-related and anti-biotic resistance genes.

*pilA, pilB, efaA_fm_* and *esp* genes showed a strong correlation with the biofilm producing capacity of the isolates, but *gelE, fsr* and *hyl* genes were present in a low number of biofilm-producing *E. faecium* isolates. Among the antibiotic resistance genes, *pbp5, vanA* and *pbpZ* genes had the highest frequency, *vanB* was the least frequent among the isolates, and none were positive for the *blaZ* gene (Table 4). Furthermore, a low number of biofilm producing *E. faecium* isolates harbored the *fsr* gene and none of those isolated from blood and catheter amplified the gene. On the other hand, most of the isolates were found to be *pilA, pilB* and *esp*-positive compared to the non-biofilm-forming isolates. When comparing the isolates based on their origin, 21% of urine isolates, 8% of fecal isolates, and 12% of blood isolates were either strong or weakly adherent biofilm producers (Table 3).

**Table 4:** Virulence and resistance genes among biofilm positive and biofilm negative isolates

**Virulence genes**	**Clinical Isolates**		**Environmental Isolates**	

	**Biofilm Positive**	**Biofilm Negative**	**Biofilm Positive**	**Biofilm Negative**
*pilA/pilB/efaA_fm_/esp/hyl/asa1/gelE*	5	4	0	0
*pilA/pilB/efaA_fm_/esp/hyl/gelE*	2	3	0	0
*pilA/pilB/efaA_fm_/hyl/asa1/fsr*	10	6	0	0
*pilA/pilB/esp/hyl*	25	6	6	5
*pilA/pilB/efaA_fm_/esp*	67	10	17	15
*hyl/gelE/fsr*	5	14	14	20
*pilA/pilB/efaA_fm_*	36	3	0	0
*pilA/pilB*	18	11	6	0
**Resistance genes**				
*pbp5/vanA /pbpZ*	64	8	10	2
*vanB*	18	3	4	0
The values are presented as percent.				

### The PFGE analysis.

**Fig. 3:**
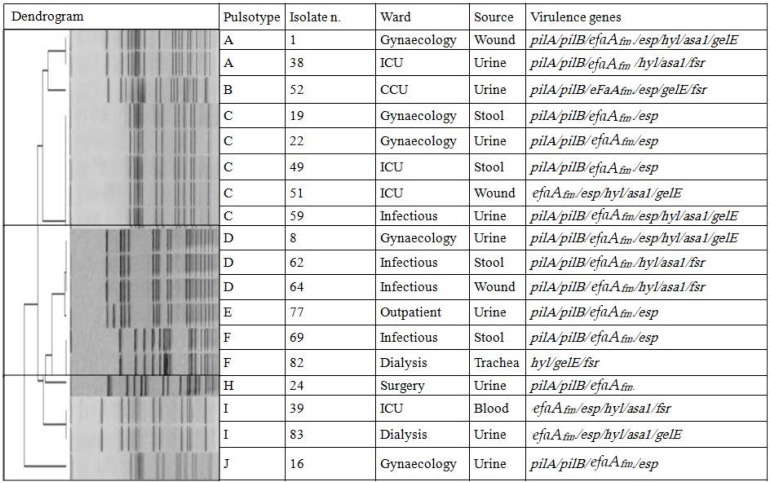
Dendrogram cluster analysis of PFGE data for 18 biofilm-producing *E. faecium* isolates with vancomycin/penicillin/gentamycin resistance phenotypes

**Fig. 4:**
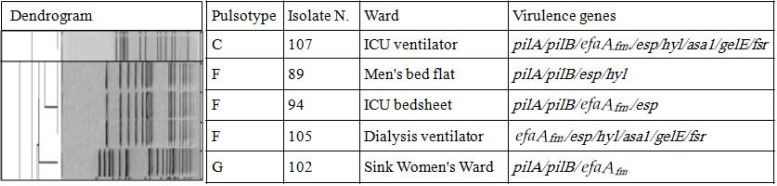
Dendrogram cluster analysis of PFGE data for 5 environmental biofilm-producing *E. faecium* isolates with vancomycin/penicillin/gentamycin resistance phenotypes

PFGE was done to perform the genetic linkage analysis among the clinical and environmental isolates. These isolates were classified into 10 pulsotypes (A–J) by considering a similarity cutoff ≥ 95%. The predominant pulsotype (C) comprised of 6 isolates (26%); 5 isolates (2.2%) showed a unique PFGE pattern as shown in Figs. 3 and 4. The C, F, and D pulsotypes with 6, 5, and 3 isolates were the most pulsotypes. The C and F pulsotypes were observed both in clinical and environmental isolates. The pulsotype G was observed only in environmental isolates. A total of 2 isolates with pulsotype I from urine and blood samples collected from ICU and dialysis wards carried the both *vanA* and *vanB* genes simultaneously with MIC of more than 1024 μg/mL for vancomycin (Fig. 3). In pulysotype C, the dominant profile of virulence genes was *pilA/pilB/efaA_fm_/esp.*

## DISCUSSION

In the present study, biofilm-producing strains among *E. faecium* clinical and environmental isolates were investigated. Clinical and environmental isolates were compared for clonal relationship and virulence determinants. In this study, clinical isolates displayed higher antibiotic resistance compared with environmental isolates. More than 50% of the clinical isolates were resistant to penicillin, ampicillin, vancomycin, and erythromycin. Environmental isolates showed low resistance to these antibiotics. Furthermore, 45% of the clinical isolates were resistant to vancomycin (VRE), whereas 6% of the environmental isolates showed this resistance. Other investigators have found various *E. faecium* resistance rates to vancomycin up to 11% in Europe or 17% in Nigeria (15). Studies in Iran have shown an increasing trend in the incidence of resistance to vancomycin in recent years. In agreement to our study, Kafil et al. reported 23% (16), Teymournezhad et al. 25%, Hosseini et al. 14.6%, and Shokohi et al. 49% vancomycin resistance in *E. faecium* isolates collected from clinical specimens in Iranian hospitals (17).

We aslo investigated bacterial biofilm formation and observed greater biofilm production among strains isolated from urine samples than those isolated from other samples (Table 3). Quantitative evaluation of biofilm formation revealed that 49% and 33% of all vancomycin resistance and vancomycin susceptible isolates could produce biofilms, respectively. This finding was lower than reports by other investigators. In Japan 63% (18), in Spain 62% (19), and in the USA 79% (20) of the *E. faecium* isolates from clinical samples were found to form biofilms.

A number of studies showed an association between virulence genes and biofilm formation. Moreover, 84% of biofilm-producing *E. faecium* isolates have *pilA, pilB, efaA_fm_* and *esp* genes. In non-biofilm-producing *E. faecium* isolates, the *gelE, fsr* and *hyl* genes were found to be higher than other genes. In most of biofilm producing *E. faecium* isolates, at least *vanA* or *vanB* genes were detected, however, a small number of non-producing *E. faecium* isolates have these resistance genes. The results of this study were in agreement with those of the study by Sharifi et al. in 2012 (21) and Kafil et al. in 2013 (22) but was in contrast to the results of Kashef et al. in 2017 (23).

Studies conducted in Iranian hospitals on the distribution of *E. faecium* have found the polyclonal strains to be dominant among clinical isolates (similar to reports from Saudi Arabian hospitals). However, reports from the USA and Europe have shown clonal spreading. In studies conducted in Iran by Pour-Shafi et al. in 2008, 34 clones (24), by Talebi et al. in 2008, 44 clones (25), by Jahangiri et al. in 2010, 29 clones (26), and by Shokohi et al. in 2013, 17 clones (27) were reported, which were consistent with our results. The specific PFGE patterns among the studied isolates demonstrates the presence of *E. faecium* strains with similar clone types in each of the hospitals, ie, pulsotypes C in Shahid Chamran hospital and pulsotype A in Shariati hospital were identified. The pulsotype patterns suggest that there was an inter hospital dissemination of pulsotype F and D. Common pulsotype (C and F) were observed among strains isolated from the patients and hospital environment (Fig. 3). These results were consistent with those of Shokohi et al. in 2013 (27).

In conclusion, our data demonstrated that biofilm-producing *E. faecium* strains were more resistant to antibiotics than non-biofilm-producing strains and were characterized by more virulence and resistance genes. The significant similarity found in the incidence of antibiotic resistance, virulence factors, and PFGE types suggest a common source of isolates from patients and environmental isolates. Increasing resistance rate of *E. faecium* to most common antibiotics calls for applying more preventive and control measures.
